# p.His16Arg of STXBP1 (MUNC18-1) Associated With Syntaxin 3B Causes Autosomal Dominant Congenital Nystagmus

**DOI:** 10.3389/fcell.2020.591781

**Published:** 2020-11-04

**Authors:** Yulei Li, Lei Jiang, Lejin Wang, Cheng Wang, Chunjie Liu, Anyuan Guo, Mugen Liu, Luoying Zhang, Cong Ma, Xianqin Zhang, Shangbang Gao, Jing Yu Liu

**Affiliations:** ^1^Key Laboratory of Molecular Biophysics of the Ministry of Education, College of Life Science and Technology, Huazhong University of Science and Technology, Wuhan, China; ^2^School of Basic Medical Sciences, Hubei University of Medicine, Shiyan, China; ^3^Department of Ophthalmology, Peking University People’s Hospital, Beijing, China; ^4^Institute of Neuroscience, State Key Laboratory of Neuroscience, CAS Center for Excellence in Brain Science and Intelligence Technology, Chinese Academy of Sciences, Shanghai, China

**Keywords:** autosomal dominant congenital nystagmus, STXBP1/MUNC18-1, syntaxin 3B, *Caenorhabditis elegans*, neurotransmitter release

## Abstract

Congenital nystagmus (CN) is an ocular movement disorder manifested as involuntary conjugated binocular oscillation and usually occurs in early infancy. The pathological mechanism underlying CN is still poorly understood. We mapped a novel genetic locus 9q33.1-q34.2 in a larger Chinese family with autosomal dominant CN and identified a variant (c.47A>G/p.His16Arg) of *STXBP1* by exome sequencing, which fully co-segregated with the nystagmus phenotype in this family and was absent in 571 healthy unrelated individuals. The *STXBP1* encodes syntaxin binding protein 1 (also known as MUNC18-1), which plays a pivotal role in neurotransmitter release. In *unc-18* (nematode homolog of *MUNC18-1*) null *Caenorhabditis elegans*, we found that the p.His16Arg exhibits a compromised ability to rescue the locomotion defect and aldicarb sensitivity, indicating a functional defect in neurotransmitter release. In addition, we also found an enhanced binding of the p.His16Arg mutant to syntaxin 3B, which is a homolog of syntaxin 1A and specifically located in retinal ribbon synapses. We hypothesize that the variant p.His16Arg of STXBP1 is likely to affect neurotransmitter release in the retina, which may be the underlying etiology of CN in this family. Our results provide a new perspective on understanding the molecular mechanism of CN.

## Introduction

Congenital nystagmus (CN, OMIM 310700) is the involuntary oscillation of eyes, a common ocular disorder usually accompanied by reduced visual acuity, head nodding, strabismus and abnormal head position (Liu et al., 2007). It appears at birth or within the first few months of life, and often occurs in isolation or coupled with other visual diseases such as albinism, congenital cataracts, aniridia, or optic nerve hypoplasia (Gottlob and Proudlock, 2014). The directions of eye oscillations include horizontal, vertical, rotatory, or combinations of these directions, with horizontal being the most common. The prevalence of all forms of CN is estimated to be 0.14% in Western countries (Sarvananthan et al., 2009), while it is 0.025% in Chinese population (Hu, 1987). It is worth noting that CN is often recognized as a unique phenotype of patients with congenital stationary night blindness (CSNB) and foveal hypoplasia due to the lack of visual electrophysiological and optical coherence tomography (OCT) examinations (Roussi et al., 2011; Thomas et al., 2014).

The models of inheritance for CN are autosomal dominant, autosomal recessive, X-linked dominant and X-linked recessive. Of these, X-linked inheritance is the most common form. Up to now, three loci for X-linked CN have been mapped to Xq26.3-q27.1 (NYS1, OMIM 310700) (Kerrison et al., 1999), Xp11.4-p11.3 (NYS5, OMIM 300589) (Cabot et al., 1999) and Xp22 (NYS6, OMIM 300814) (Liu et al., 2007), and four loci for autosomal dominant congenital nystagmus (AD-CN) have been identified on 6p12 (NYS2, OMIM 164100) (Kerrison et al., 1996), 7p11.2 (NYS3, OMIM 608345) (Klein et al., 1998), 13q31-q33 (NYS4, OMIM 193003) (Ragge et al., 2003) and 1q31.3-q32.1 (NYS7, OMIM 614826) (Xiao et al., 2012). So far, only four pathogenic genes of CN have been identified. *FRMD7* (OMIM 300628) has been linked to NYS1 (Tarpey et al., 2006), and *GRP143* (OMIM 300808) has been associated to NYS6 (Liu et al., 2007). Recently, mutations in *MANBA* (OMIM 609489) and *AHR* (OMIM 600253) has been reported to cause autosomal dominant CN (Yu et al., 2015) and autosomal recessive CN (Mayer et al., 2019), respectively. Furthermore, structural variations in a non-coding region within the NYS7 locus have been linked to autosomal dominant CN (Sun et al., 2020). However, the role of these genes in CN is still largely unknown. Although several hypotheses have been proposed to elucidate the neurological mechanisms underlying CN, there is still no consensus on the pathogenesis of CN. Therefore, identification of more pathogenic genes and further exploration of their physiological function will advance our understanding of the etiology and pathogenesis of nystagmus.

*STXBP1* (OMIM 602926) encodes syntaxin binding protein 1, commonly known as MUNC18-1, which plays a critical role in neurotransmitter release (Ma et al., 2013). Pathogenic variants in *STXBP1* have been reported to be mainly involved in epileptic encephalopathy, early infantile, 4 (EIEE4, OMIM 612164) with or without nystagmus (Saitsu et al., 2008; Stamberger et al., 2016). The interactions between MUNC18-1 and different syntaxin proteins are essential for membrane fusion and neurotransmitter release in various tissues. Syntaxin 1B is a syntaxin protein mostly expressed in central nervous system and pathogenic variants in which cause generalized epilepsy with febrile seizures plus, type 9 (OMIM 616172), with nystagmus symptom was noted in some patients (Vlaskamp et al., 2016). Epithelial cells and nervous system expressed *STX3* (OMIM 600876) has recently been reported as the pathogenic gene of microvillus inclusion disease (MVID) with or without nystagmus (Julia et al., 2019). Furthermore, gabapentin (analog of inhibitory neurotransmitter gamma-aminobutyric acid) and memantine (antagonist of excitatory neurotransmitters) were reported as effective drugs for the treatment of CN (McLean et al., 2007). These studies raised the question whether synaptic transmission plays a pivotal role in the pathogenesis of CN.

In this study, we mapped a novel genetic locus 9q31.3-q34.2 of AD-CN and identified a variant (c.47A>G/p.His16Arg) of *STXBP1* in a large Chinese family with CN and visual electrophysiological abnormalities. We found that the p.His16Arg mutant results in reduced locomotion and aldicarb sensitivity in *unc-18* (nematode homolog of *MUNC18-1*) null *Caenorhabditis elegans*, and exhibits a stronger binding to syntaxin 3B. We hypothesize that the variant p.His16Arg of MUNC18-1 may affect neurotransmitter secretion in the retina, which is the underlying etiology of CN. Our findings provide a new perspective on the significance of synaptic transmission underlying the pathological mechanism of nystagmus.

## Materials and Methods

### Patients and Genomic DNA Extraction

A large Chinese family from Ningxia Autonomous Region with CN was identified and characterized at Peking University People’s Hospital. The study was approved by the ethics committee of Huazhong University of Science and Technology (Wuhan, China) and conformed to the Declaration of Helsinki. Informed consents and 5 ml peripheral blood samples were obtained from eight affected and fifteen unaffected family members. Genomic DNA was extracted according to standard procedures using the Promega Wizard Genomic DNA Purification Kit (Promega, United States).

### Linkage and Genotyping Analysis

Genome-wide linkage scan of this family was carried out with 382 fluorescent microsatellite markers from ABI Mapping Panel MD-10 (Applied Biosystems, United States). Microsatellite markers were genotyped using an ABI 3730 Genetic Analyzer (Applied Biosystems, United States). Genotypes were analyzed by the GeneMapper 2 Software (Applied Biosystems, United States). Two-point linkage analysis was performed as previously described (Dai et al., 2008). Microsatellite markers for fine mapping on chromosome 9 were obtained from the Marshfield Clinic Medical Genetics database.

### Exome Sequencing and Sanger Sequencing

Exome sequencing was conducted by BGI Genomics (BGI, China). Detail methods were described previously (Zhang et al., 2013). The variants of candidate genes were verified by Sanger sequencing. Primers used to identify and analyze the *STXBP1* variant are listed in Supplementary Table S2.

### Restriction Fragment Length Polymorphism (RFLP) Analysis

The c.47A>G variant of *STXBP1* would result in a loss of the *Nsi*I restriction site. Therefore, PCR primers designed for RFLP analysis are listed in Supplementary Table S3. The PCR products were digested with *Nsi*I (New England Biolabs, United States) overnight at 37°C and separated on 2% agarose gels to classify the wild-type and mutant alleles.

### RT-PCR and Plasmid Construction

Total RNA was isolated from SH-SY5Y cell line or mouse tissue or worms with TRIzol reagent (Invitrogen, United States) according to the manufacturer’s protocol. cDNA was generated using the M-MLV RT-PCR kit (Invitrogen, United States) with 1 μg of total RNA from each sample. Expression of mRNA in worms was analyzed with ABI 7900HT Real-Time PCR system (Applied Biosystems, United States) using SYBR Green mix (Roche Diagnostics, Germany). Primers used for qRT-PCR are listed in Supplementary Table S3.

The full-length coding sequence of human *STXBP1* (ENST00000373299) and *STX3B* (ENST00000633708) were obtained from SH-SY5Y cells cDNA, while the CDS of mouse *Stxbp1* (ENSMUST00000050000) and *Stx3B* (ENSMUST000000 47698) were obtained from mouse retina cDNA. All the missense mutants were introduced by Overlap-PCR based on wild-type’s sequence.

### Cell Culture and Transfection

Neuro-2A cells, SH-SY5Y cells and HeLa cells were cultured in Dulbecco’s modified Eagle medium (Gibco, United States) supplemented with 10% fetal bovine serum (Gibco, United States) at 37°C and 5% CO_2_. Cells were transiently transfected using Lipofectamine 2000 (Invitrogen, United States) in Opti-MEM I Reduced Serum (Gibco, United States), according to the manufacturer’s instructions. After transfection for 24 h or 36 h, cells were harvested for RNA/protein extraction or immunofluorescent labeling.

### Western Blot, GST Pull-Down and Co-immunoprecipitation

Western blot, GST pull-down and co-immunoprecipitation assays were performed as previously described (Huang et al., 2015). Flag tagged human MUNC18-1, Flag tagged human syntaxin 1A (STX1A), Flag tagged human syntaxin 3B (STX3B), and Flag tagged mouse syntaxin 3B (Stx3B) were extracted from HeLa cells transfected with corresponding plasmids. GFP tagged human MUNC18-1 and GFP tagged worm UNC-64 were extracted from HeLa cells transfected with corresponding plasmids. GST tagged human MUNC18-1 was extracted from *Escherichia coli* Rosetta strain transformed with human MUNC18-1 cDNA plasmids. Mouse syntaxin 3 (Stx3) was extracted from mouse retina lysate. The primary antibodies were as follows: MUNC18-1 (1:500, mouse monoclonal antibody, BD Biosciences, Canada), Syntaxin 3 (1:1000, rabbit polyclonal antibody, Abcam, United Kingdom), GFP (1:5000, rabbit polyclonal antibody, Proteintech, United States), FLAG (1:5000, mouse monoclonal antibody, MBL, Japan), GPADH (1:3000, mouse monoclonal antibody, Proteintech, United States), GST (1:5000, rabbit polyclonal antibody, ABclonal, United States), beta-actin (1:3000, mouse monoclonal antibody, CST, United States). Quantitative analysis of protein bands was performed by the Image J software^1^.

### Preparation of Frozen Sections and Immunofluorescent Labeling

The mouse eyes were rapidly enucleated after cardiac perfusion and were incubated immediately in 4% paraformaldehyde for 30 min at room temperature. The eyes were equilibrated in 30% sucrose overnight followed by washing in PBS three times, and then embedded in optical cutting technology freezing medium and fast frozen. Sections were cut on Leica CM1950 cryostat (Leica, Germany) at 10 μm thickness and collected on the gelatin-coated slides.

Immunofluorescence assay was performed as previously described (Liu et al., 2015). The primary antibodies were as follows: MUNC18-1 (1:50, mouse monoclonal antibody, BD Biosciences, Canada), Syntaxin 3 (1:100, rabbit polyclonal antibody, Abcam, United Kingdom), FLAG (1:500, mouse monoclonal antibody, MBL, Japan).

### Mice

This study was conducted using adult C57BL/6J mice. All animal procedures were approved by Institutional Animal Care and Use Committee at Huazhong University of Science and Technology.

### Worm Assays

N2 (wild-type), CB81 [*unc-18* (*e81*)], CB234 [*unc-18* (*e234*)], NM204 [*snt-1* (*md290*)] and VC223 [*tom-1* (*ok285*)] strains were obtained from the Caenorhabditis Genetics Center (CGC, United States). All the worms were cultured at 22°C as described previously (Brenner, 1974).

Transgenic worms were generated according to the standard microinjection procedures (Mello et al., 1991). Human *STXBP1* cDNA and *C. elegans unc-18* cDNA were cloned into pPD95.75 vector which contains a 2.0 kb promoter of *snb-1* gene and a GFP tag. In co-expression experiments, wild-type human STXBP1 protein was fused with the GFP tag, while the p.His16Arg mutant STXBP1 protein was fused with the RFP tag. Expressing vectors were injected at 50 ng/μl together with P*lin44*::GFP (5 ng/μl) as a co-injection marker. Worms which showed green fluorescence in the neurons and tail (P*lin44*::GFP) were selected and bred into a non-integrated line. In wild-type and p.His16Arg mutant *STXBP1* co-expressing worms, only worms which showed both green and red fluorescence in the neurons were selected. At least five independent non-integrated lines were examined for rescue experiments.

Locomotion behavioral assay was performed as follows. Young adult nematodes were placed on nematode growth medium (NGM) plates contain OP50 *E. coli* lawn. The animals were allowed to rest and adapt to the new NGM plates for 10–15 min before recording. One-minute locomotion of worms was screened under a Zeiss Discovery V8 stereomicroscope (Carl Zeiss MicroImaging Gmbh, Germany), and the image sequences were captured with an Andor iXonEM + DV885K EMCCD camera (Andor, United Kingdom). The average movement rate (pix/s) were analyzed and calculated with Multi-Worm Tracker software (Swierczek et al., 2011).

Aldicarb stimulation assay was performed as follows. Aldicarb (Sigma Aldrich, United States) was dissolved in 70% ethanol to prepare 100 mM aldicarb stock solution and stored in −20°C. The 0.5 mM aldicarb NGM plates were prepared fresh two days before each experiment for appropriate moisture. Control and rescue lines were examined simultaneously under the same condition. Young adult worms were transferred to the 0.5 mM aldicarb NGM plates seeded with OP50 *E. coli* lawn. Worms were considered paralyzed if they failed to response to the stab of a platinum filament thrice. About 25 worms were picked up to aldicarb containing NGM plates in each individual assay, and the percentage of moving animals was measured every 15 min. The aldicarb stimulation assay was carried out double-blindedly, and was performed by one person for consistent standard of paralyzed worms.

### Bioinformatics

The human homolog of mouse syntaxin 3B was searched by ensembl BLAST/BLAT search tool^2^. Protein sequences were aligned with Clustalw2 software^3^. The mouse and human syntaxin 3B protein’s sequences were aligned and displayed by using the Boxshade program^4^.

### Statistical Analyses

All data are represented as the mean ± standard deviation (SD). Comparison of means was performed using SPSS v22 for Windows (IBM, United States) evaluated using unpaired two-tailed *t*-test. The asterisk indicates statistical significance (^∗^ means *p* < 0.05, ^∗∗^ means *p* < 0.01, ^∗∗∗^ means *p* < 0.001).

## Results

### Clinical Characterization of the Patients With AD-CN

**FIGURE 1:**
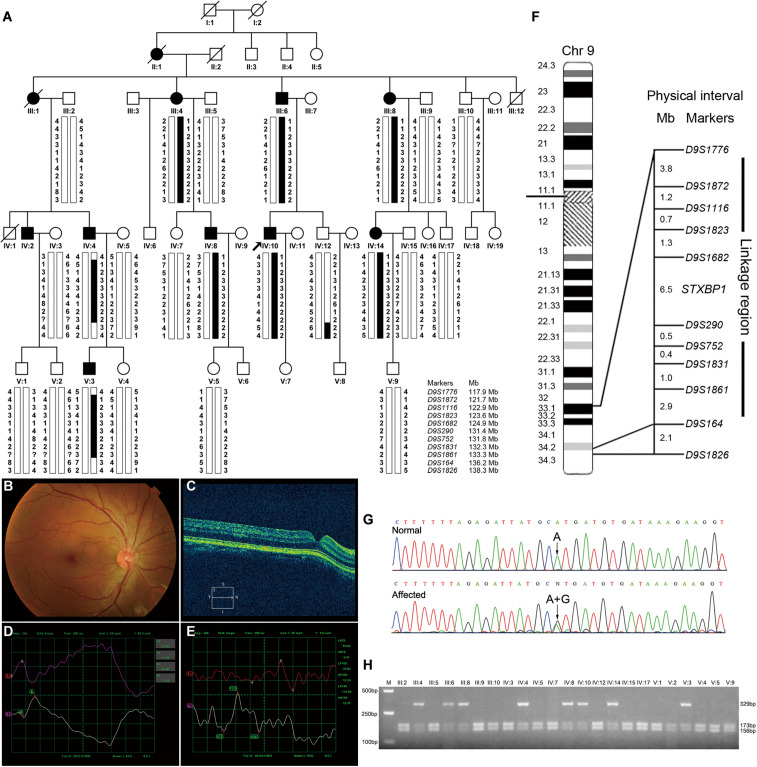
Pedigree structure, clinical characteristics of the index case, genotypic analysis, diagram of chromosome 9, identification of *STXBP1* variant and RFLP analysis of the AD-CN family. **(A)** The pedigree structure and microsatellite haplotype analysis of the Chinese family with AD-CN. Individuals with CN are indicated by solid squares (males) or solid circles (females). Unaffected individuals are indicated by open symbols. Deceased individuals are indicated by slashes (/). The proband is indicated by an arrow. The disease haplotype is shown in black vertical bar; the variants of eight microsatellite markers (*D9S1872*, *D9S1116*, *D9S1823*, *D9S1862*, *D9S290*, *D9S752*, *D9S1831*, and *D9S1861*) are co-segregated with the clinical manifestation in the family. **(B,C)** Normal fundus **(B)** and normal foveal **(C)** of the proband. **(D)** The flash electroretinogram (fERG) of the proband. Both eyes show shortened latency and decreased amplitude of b-wave. The b-wave latency of right eye and left eye are 18.5 ms and 41.0 ms, respectively. The amplitudes of b-wave of right eye and left eye are 52.25 μV and 71.29 μV, respectively. **(E)** The pattern visual evoked potential (PVEP) of the proband. The amplitude of P100 wave in both eyes was decreased. The right eye shows a delayed latency of P100 wave, while the left eye shows a shortened latency of P100 wave. The amplitudes of P100 wave of right eye and left eye are 7.71 μV and 13.24 μV, respectively. The latency of P100 wave of right eye and left eye are 154 ms and 78.5 ms, respectively. **(F)** The linkage interval and flanking microsatellite markers are indicated. **(G)** DNA sequencing chromatograms show the heterozygous variant in *STXBP1* identified in this study. **(H)** The *Nsi*I restriction analysis showing full segregation of the c.47A>G variant with the disease phenotype in the family.

The proband (Figure 1A, IV-10) was a 30-year-old male patient who suffered from conjugated pendular nystagmus (Supplementary Video S1) accompanied with astigmatism and amblyopia. The nystagmus was intensified when he gazed at objects or followed with eye fatigue, and was diminished when he closed his eyes. His visual acuity of the right eye and left eye was 20/200 and 20/100, respectively. The ocular fundus revealed normal appearance of the optic nerve heads, blood vessel arrangement and retina pigmentation (Figure 1B). The well-formed fovea was shown by OCT (Figure 1C). The vestibular function of the proband was normal, with no photophobia, night blindness, color blindness or neurological symptoms observed (data not shown). Binocular flash electroretinograms (fERG) revealed that b wave is ahead with lower amplitude (Figure 1D). Pattern visual evoked potential (PVEP) showed delayed latency with low amplitude of P100 wave in the right optic nerve and shortened latency with low amplitude of P100 wave in the left optic nerve (Figure 1E).

Patient III-4 suffered from rotary nystagmus (Supplementary Video S2), and his visual acuity was 20/100 in both eyes. Patient III-6 showed horizontals jerk nystagmus (Supplementary Video S3) with orbicularis oculi muscle spasm, and patient III-8 had jerk horizontal nystagmus (Supplementary Video S4) with astigmatism and poor binocular visual acuity. Both eyes of patient IV-8 exhibited major jerk nystagmus (Supplementary Video S5) with restricted abduction of the right eye, and the visual acuity of both eyes were 20/200. Patient IV-14 displayed mixed pattern nystagmus (Supplementary Video S6) with low amplitude, poor binocular visual acuity and astigmatism. Patient V-3 exhibited pendular nystagmus (Supplementary Video S7) and poor binocular visual acuity. No patient showed progressive visual loss or aggravated oscillation of the eyes in the family, and no other ophthalmological or neurological abnormalities were observed.

### Mapping of a Novel Genetic Locus for AD-CN on Chromosome 9q33.1-q34.2

At present, four genetic loci of AD-CN (6p12, 7p11.2, 13q31-q33, and 1q31.3-q32.1) have been reported. We therefore firstly genotyped the Chinese AD-CN family with twenty-one markers flanking above known AD-CN genetic loci. Linkage analysis showed negative LOD (log of the odds ratio) scores at a recombination fraction of zero for all twenty-one markers (data not shown), thus excluded the four known AD-CN loci. Recently, pathogenic variants of *MANBA* associated with AD-CN have been reported (Yu et al., 2015), so we performed direct sequencing analysis of *MANBA* gene in the proband, but no pathogenic variant was found.

To identify the pathogenic gene responsible for the Chinese family with AD-CN, we next undertook a genome-wide linkage scan with microsatellite markers in the family (including 8 patients and 15 unaffected individuals) as well as follow-up fine mapping. Our results showed a positive linkage with markers (*D9S1872, D9S1116, D9S1823, D9S1682, D9S290, D9S752, D9S1831*, and *D9S1861*) on chromosome 9 (Figure 1A). The LOD scores for the markers in chromosome 9 are shown in Supplementary Table S1. Patients IV-4 and V-3 displayed recombination events between markers *D9S1776* and *D9S1872*. Patients IV-4, V-3 and normal individual IV-12 showed recombination events between markers *D9S1861* and *D9S164*. Four markers (*D9S1872, D9S1116, D9S290*, and *D9S752*) generated LOD scores greater than 3, the cut-off LOD score for significant linkage. These results suggest that the gene responsible for AD-CN in this family lies between *D9S1776* and *D9S164* on chromosome 9q33.1-q34.2, a genomic region of 18.3 Mb (Figure 1F).

### Identification of a Novel Variant c.47A>G/p.His16Arg of STXBP1 Responsible for AD-CN

The mapped interval spans 18.3 Mb and contains 202 protein-coding genes. Three genes involved in neural development and regulation of neural activity, *LHX2* (OMIM 603759), *LHX6* (OMIM 608215) and *FREQ* (OMIM 603315), were directly sequenced in the proband but failed to identify a pathogenic variant responsible for CN. To identify the pathogenic variants, genomic DNA from the proband was sent to BGI Genomics for exome sequencing. The summary of exome sequencing data is shown in Supplementary Table S4. Non-synonymous, splicing and indel variants with a minor allele frequency no more than 0.01 were filtered against dbSNP and gnomAD databases to exclude polymorphisms. Direct Sanger sequencing was performed on the exons of low coverage in the linked region (Supplementary Table S5), and the variants detected in the interval are listed in Supplementary Table S6.

After detailed analysis of all variants within the linked interval, a variant (c.47A > G) in *STXBP1* was identified (Figure 1G). This variant is predicted to result in a histidine to arginine substitution at amino acid residue 16 (p.His16Arg) located in domain 1 of STXBP1 (Supplementary Figure S1A). PCR-RFLP assay revealed that this variant fully segregated with the nystagmus phenotype in the family (Figure 1H), but was not found in 571 unrelated healthy Chinese Han individuals (data not shown). It is noteworthy that the variant c.47A>G is a polymorphism (rs571127140) in dbSNP database. Further analysis showed that such a polymorphism arises from Genome Aggregation Database (gnomAD), and its allele frequency is 1/251,356 in the database. The variation carrier came from the Human Genome Diversity Panel (HGDP), an anonymous collection of globally diverse DNA samples gathered many years ago (personal communication with the variation submitter), which cannot be traced at present, and it is impossible to ascertain whether the variation carrier was affected by CN. The p.His16Arg variant was predicted to be deleterious by MutationTaster (score at 29) and CADD (score at 23.2), demonstrating an evolutionally conserved His16 residue of MUNC18-1 from *Xenopus tropicalis* to *Homo sapiens* (Supplementary Figure S1B).

### The Variant p.His16Arg of MUNC18-1 Impairs Neurotransmitter Release in *C. elegans*

**FIGURE 2:**
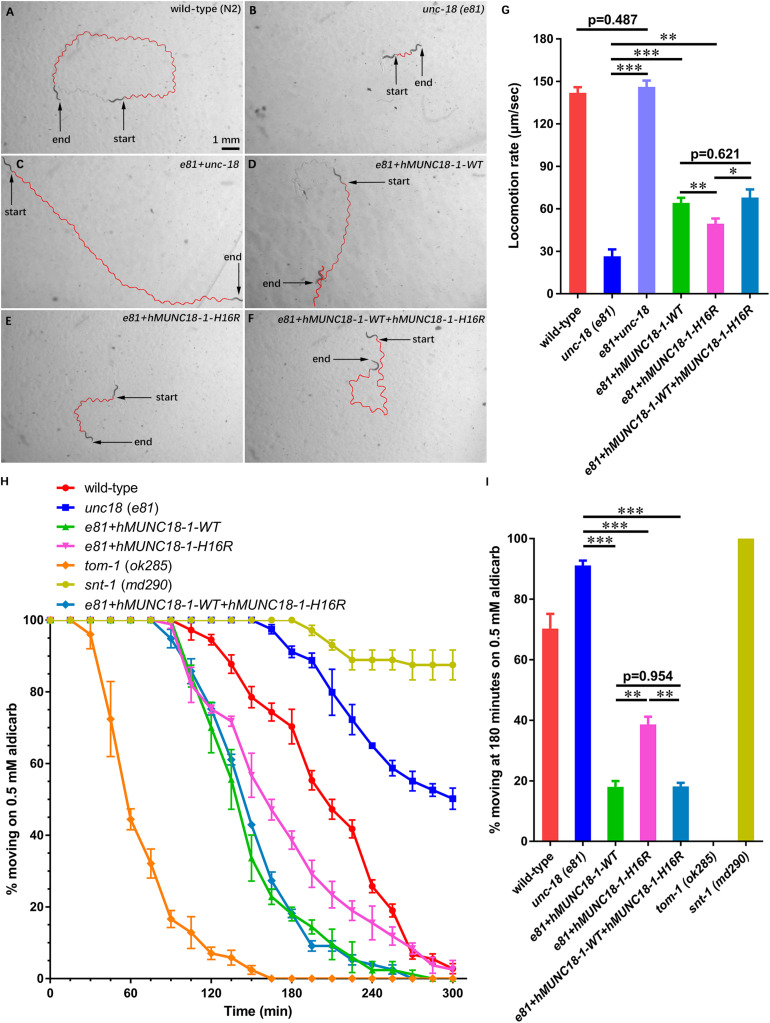
Human MUNC18-1 transgenic *unc-18* (*e81*) *C. elegans* altered their locomotion and aldicarb resistance. **(A–F)** The locomotion trail of wild-type **(A)**, *unc-18* (*e81*) **(B)**, *e81* + *unc-18* **(C),** *e81* + *hMUNC18-1*-WT **(D),** *e81* + *hMUNC18-1*-H16R **(E)** and *e81* + *hMUNC18-1*-WT + *hMUNC18-1*-H16R **(F)** worms on NGM plate in 1 min. The traces of worms’ locomotion in 1 min are outlined in red, start and end represent the start and end position of individual worm in 1-min recording, respectively. **(G)** The average locomotion rate (μm/sec) of wild-type (*n* = 16), *unc-18* (*e81*) (*n* = 15), *e81* + *unc-18* (*n* = 17), *e81* + *hMUNC18-1*-WT (*n* = 33), *e81* + *hMUNC18-1*-H16R (*n* = 35), and *e81* + *hMUNC18-1*-WT + *hMUNC18-1*-H16R (*n* = 19) strains. * means *p* < 0.05, ** means *p* < 0.01, *** means *p* < 0.001. **(H)** The paralysis time-course of *unc-18* (*e81*), *e81* + *unc-18, e81* + *hMUNC18-1*-WT, *e81* + *hMUNC18-1*-H16R and *e81* + *hMUNC18-1*-WT + *hMUNC18-1*-H16R worms on NGM plate containing 0.5 mM aldicarb (*n* = 3 repeats). *tom-1* (*ok285*) strain and *snt-1* (*md290*) strain are used as hypersensitivity control and super-resistance control, respectively. **(I)** The survival ratio of 7 worm strains on NGM plate containing 0.5 mM aldicarb at 180 min (*n* = 3 repeats), ** means *p* < 0.01, *** means *p* < 0.001.

*STXBP1* encodes the syntaxin binding protein 1 MUNC18-1, pathogenic variants of which has been reported to be associated with EIEE4 (Saitsu et al., 2008). MUNC18-1 plays a pivotal role in soluble *N*-ethyl maleimide sensitive-factor attachment protein receptors attachment protein receptor (SNARE) complex assembly and synaptic transmission (Sudhof and Rizo, 2011; Ma et al., 2013). To analyze whether p.His16Arg affects the function of MUNC18-1 in neurotransmitter release (especially the excitatory neurotransmitter acetylcholine at neuromuscular junction), we set out to test this using *C. elegans* as a model organism, as there are high sequence conservation and functional homology between *C. elegans* UNC-18 and human MUNC18-1 (hMUNC18-1) proteins (The human MUNC18-1 protein shared an identity of 59% and a positivity of 75% with the *C. elegans* UNC-18 protein, while the corresponding position of human MUNC18-1 protein His16 residue in *C. elegans* UNC-18 protein is Asn13, which were showed in Supplementary Figure S2), and as that simple behavioral assays of *C. elegans* have facilitated the research of proteins involved in synaptic function (Gengyo-Ando et al., 1996). Our data revealed that the *unc-18* deficient worms exhibit severe uncoordination (Figures 2A,B,G), which is similar to the previous report (Weimer et al., 2003). *e81* worms expressing the *C. elegans* autologous *unc-18* showed similar locomotion rate as wild-type (N2) worms (Figure 2A,C,G), demonstrating a reliable approach. Both hMUNC18-1 WT and p.His16Arg mutant can partially rescue the locomotion defect of *e81* null worms, but the *e81* worms which expressing p.His16Arg mutant showed a weaker locomotion than those expressing WT (Figures 2B,D,E,G). We also conducted the locomotion assays in *e81* worms co-expressing wild-type hMUNC18-1 and p.His16Arg mutant at a 1:1 ratio. These worms exhibited a similar motor ability as *e81* worms expressing only the wild-type hMUNC18-1 (Figures 2D,F,G). The movements of individual worm in each strain (including N2, *e81*, *e81* + *unc-18*, *e81* + hMUNC18-1-WT, *e81* + hMUNC18-1-H16R and *e81* + hMUNC18-1-WT + hMUNC18-1-H16R) were recorded as shown in Supplementary Videos S8–13. Similar results were found in *e234* worms rescued by MUNC18-1 WT and p.His16Arg mutant (Supplementary Figures S3A–E). These findings suggest a potential effect of p.His16Arg mutant on synaptic function.

Exposing worms to aldicarb (acetylcholinesterase inhibitor) causes paralysis, due to the inability to turn off acetylcholine signaling at the neuromuscular junction which leads to hypercontraction (Mahoney et al., 2006). The hMUNC18-1 WT transgenic worms showed higher aldicarb sensitivity than p.His16Arg mutant expressing worms at 180 min (Figures 2H,I). *e81* worms co-expressing wild-type and p.His16Arg hMUNC18-1 showed no obvious difference on aldicarb resistance compared to *e81* worms expressing only the wild-type hMUNC18-1 (Figures 2H,I). Moreover, we obtained similar results when using *e234* worms. The only difference is that the p.His16Arg mutant transgenic *e234* worms have no obviously enhanced aldicarb sensitivity compared with *e234* worms (Supplementary Figures S3F,G). These data suggested that the p.His16Arg mutant might cause a decrease in the total amounts of acetylcholine release in *C. elegans*.

**FIGURE 3:**
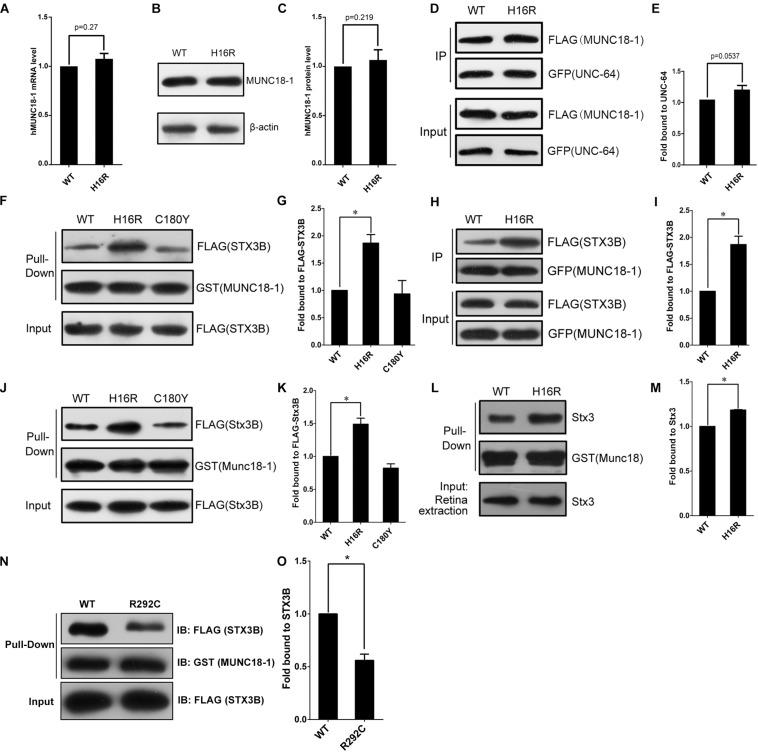
The MUNC18-1 p.His16Arg mutant shows altered interaction with human and mouse syntaxin 3. **(A)** Wild-type and p.His16Arg mutant hMUNC18-1 transgenic *e81* worms show similar *hMUNC18-1* mRNA expression levels detected by RT-qPCR (*n* = 3, *p* = 0.27). **(B,C)** Western blot demonstrates similar levels of WT and p.His16Arg mutant hMUNC18-1-GFP fusion protein in transgenic *e81* worms (*n* = 3, *p* = 0.219). **(D,E)** Co-immunoprecipitation assay of FLAG-tagged WT and mutant MUNC18-1 with GFP-fused UNC-64, p.His16Arg mutant protein shows slight enhanced binding of to UNC-64 compared with WT (*n* = 5, *p* = 0.0537). The data are represented as the mean ± SD. Statistical significance was evaluated using two-tailed *t*-test. **(F,G)** GST pull-down assay of GST-tagged wild-type and mutant MUNC18-1 with the FLAG-tagged human STX3B. The p.His16Arg mutant exhibits obviously enhanced affinity for STX3B compared with WT, while the p.C180Y mutant shows similar affinity compared with WT. The experiments were repeated 3 times independently, * means *p* < 0.05. **(H,I)** GFP tagged wild-type and p.His16Arg mutant MUNC18-1 were co-transfected into HeLa cells with FLAG-tagged human STX3B. Cell extracts were immunoprecipitated with anti-GFP antibody and analyzed by immunoblotting with anti-FLAG antibody. The p.His16Arg mutant shows overt intensive interaction with human STX3B compared with WT. The assays were repeated 3 times independently, * means *p* < 0.05. **(J,L)** GST pull-down assay of GST-tagged mouse WT and mutant Munc18-1 with the FLAG-tagged mouse Stx3B or endogenous Stx3 (mouse retina extracts). **(K,M)** Quantitative analysis of the interaction between GST-Munc18-1 and FLAG-Stx3B or endogenous Stx3 based on the data shown in **(J,L)**, respectively, * means *p* < 0.05. **(N,O)** GST pull-down assays show that p.R292C mutant which cause EIEE4 associated with nystagmus exhibits decreased binding affinity to the FLAG-STX3B fusion protein compared to wild-type MUNC18-1, the assays were repeated 3 times independently, * means *p* < 0.05.

By q-PCR and Western blot assays, we observed hMUNC18-1 WT and p.His16Arg mutant exhibited similar expression levels of mRNA and protein in transgenic *e81* worms (Figures 3A–C), suggesting that the different ability to rescue locomotion defect and aldicarb resistance in *e81* worms are unrelated to hMUNC18-1 expression level.

To elucidate the potential molecular mechanism by which p.His16Arg mutant affects the acetylcholine release at neuromuscular junction in worms, we next explored whether this mutant alters the interaction with UNC-64 (the *C. elegans* homolog of human syntaxin), as the change of binding affinity between UNC-18 and UNC-64 often impacts neuronal exocytosis in *C. elegans*. Interestingly, co-immunoprecipitation assay showed a slightly enhanced interaction of MUNC18-1 p.His16Arg with UNC-64 in comparison with WT (Figures 3D,E). Based on these findings, we speculate that p.His16Arg compromises the ability of MUNC18-1 to rescue the defects of locomotion and neurotransmitter release in *C. elegans* due to its slightly enhanced interaction with UNC-64.

### The Variant p.His16Arg of MUNC18-1 Enhances the Interaction With Syntaxin 3B but Not Syntaxin 1A

Since there is a slightly enhanced interaction of MUNC18-1 p.His16Arg with UNC-64, we are interested in knowing whether this mutant affects the interaction with syntaxin 1A (encoded by *STX1A*, OMIM 186590), because it will help us to determine whether p.His16Arg mutant exerts an effect on synaptic vesicle secretion in mammalian cells (Han et al., 2011). The result showed that the p.His16Arg mutant does not affect interaction with the ‘closed’ or ‘open’ STX1A (Supplementary Figures S4A–H). We used the p.Cys180Tyr of MUNC18-1 (a pathogenic variant resulting in EIEE4) as a control and found that it showed a weak affinity to bind with the open form of STX1A (Supplementary Figures S4A,B,E,F), which is consistent with a previous report (Saitsu et al., 2008).

The attenuated amplitudes of b-wave of fERG and P100 wave of PVEP of the proband suggests a defect of neuronal signal transduction in the retina, therefore we focus on anther protein, syntaxin 3B (encoded by *STX3*), validated as the specific SNARE molecule responsible for the exocytosis of synaptic vesicles at ribbon synapses of the rodent and goldfish retina (Curtis et al., 2010). Since the research into the human homolog of mouse syntaxin 3B has not been reported, we first aligned the mouse Stx3B to human protein database and identified an alternative transcript of human syntaxin 3 (STX3B) with 99% identity (only three amino acids difference) to mouse Stx3B (Supplementary Figure S5). Unexpectedly, we observed an enhanced interaction between the p.His16Arg mutant and STX3B, whereas the p.C180Y mutant showed no effect on this interaction (Figures 3F–I). Likewise, we observed similar results by using mouse Munc18-1 (WT and p.His16Arg mutant) and Stx3B (Figures 3J–M). Furthermore, we observed significant signals of co-localization of Munc18-1 and Stx3B within the outer plexiform layer (OPL) where ribbon synapses exist, as well as within the inner plexiform layer (IPL) (Supplementary Figure S6).

Interestingly, Stamberger et al have reported a mutation c.874C>T/p.Arg292Cys of *STXBP1* to be associated with EIEE4 and rotatory nystagmus (Stamberger et al., 2016). We found that the p.Arg292Cys mutant significantly reduced the binding of MUNC18-1 to syntaxin 3B (Figures 3N,O) by GST pull-down assay. Collectively, these findings suggest that the altered interaction between MUNC18-1 and syntaxin 3B may be linked to CN.

## Discussion

In this study, we mapped a novel AD-CN genetic locus on 9q33.1-q34.2 in a five-generation Chinese family and identified a variant (c.47A>G/p.His16Arg) of *STXBP1* within this locus. *In vitro* function analysis showed that an obviously enhanced binding of the p.His16Arg mutant to syntaxin 3B, which is a homolog of syntaxin 1A and predominantly expressed in vertebrate retina. *In vivo*, the p.His16Arg mutant exhibits a reduced ability to rescue the locomotion defect and aldicarb sensitivity in *unc-18* (nematode homolog of *STXBP1*) null *C. elegans*.

STXBP1 (MUNC18-1) plays a pivotal role in synaptic vesicle exocytosis and neurotransmitter release (Ma et al., 2013). Pathogenic variants of *STXBP1* have been associated with EIEE4 (also known as Ohtahara syndrome), which is manifested as intractable epilepsy, severe developmental delay and mental retardation (Saitsu et al., 2008; Allen et al., 2016), while nystagmus was noted in a few cases (Stamberger et al., 2016). *STXBP1* heterozygous knockout neurons demonstrated reduced synaptic vesicle release due to haploinsufficiency of *STXBP1* (Patzke et al., 2015). To determine whether this variant has effects on neurotransmitter release, we use *C. elegans* as a model to explore this issue, but the genome-edited knock-in worm cannot be generated due to the low homology at the N-terminal region between STXBP1 and UNC-18 protein. Given the *unc-18* null worms showing uncoordinated locomotion and strong resistance to aldicarb due to the severe synaptic transmission defects (particularly the decreased acetylcholine release) (Weimer et al., 2003), our findings that p.His16Arg could weaken the ability of MUNC18-1 to restore locomotion and sensibility to aldicarb of the *unc-18* null worms suggests a compromised function of this mutant in neurotransmitter release, in particular the mutant might decrease acetylcholine release at neuromuscular junction of *C. elegans* (Figures 2A–I and Supplementary Figures S3A–G). The homologous protein of human syntaxin 1A in *C. elegans* is UNC-64, which is the main target protein of UNC-18, and UNC-18 regulates *C. elegans* neurotransmitter secretion mainly through interacting with UNC-64 (Ogawa et al., 1998). Besides, the interaction between UNC-18 and UNC-13 or RAB-3 also participates in the regulation of neurotransmitter secretion (Sassa et al., 1999; Johnson et al., 2013). We tested the interaction between STXBP1 and UNC-64 and found that the p.His16Arg mutation slightly enhanced the interaction between STXBP1 and UNC-64 (Figures 3D,E). We speculate that there may be other mechanisms leading to the altered neurotransmitter secretion between wild-type and p.His16Arg mutant transgenic *e81* worms, which may involve the interaction with UNC-13 or RAB-3. In addition, the results of nematode behavioral assays using worms that co-express WT and p.His16Arg mutant hMUNC18-1 suggest that the p.His16Arg mutation most likely exerts effect via haploinsufficiency rather than a dominant-negative manner (Figures 2D,F-I). It has been reported that the impaired interactions between MUNC18-1 and STX1A inhibit vesicle docking and membrane fusion at conventional synapses (Han et al., 2011; Martin et al., 2014). Previous research (Saitsu et al., 2008) had reported that some EIEE4-causing pathogenic variants of MUNC18-1 impair the ability of the protein to bind to the open form of STX1A rather than the closed form. In our study, the p.His16Arg mutant has little effect on the interaction with either closed or open form of STX1A (Supplementary Figures S4A–H), and this helps to explain why all the affected individuals have no other neurological dysfunction such as epilepsy.

The proband showed reduced amplitudes of b-wave of fERG and P100 wave of PVEP (Figures 1D,E), suggesting an impaired neural transduction in the retina, which was consistent with the decreased acetylcholine release at the neuromuscular junction of *C. elegans* (Figures 2A–I and Supplementary Figures S3A–G). There is a specialized type of synapse in photoreceptors and bipolar cells of vertebrate retina, which is known as ribbon synapse (Morgans, 2000). Unlike conventional synapses, the retinal ribbon synapses secrete neurotransmitters continuously, and the mechanism of neurotransmitter release in retinal ribbon synapses differ from that at conventional synapses (Morgans, 2000; Curtis et al., 2010). At conventional synapses, the core SNARE complexes including synaptic vesicle protein synaptobrevin 2/VAMP2, syntaxin 1 and SNAP-25 (Sudhof and Rizo, 2011), while at ribbon synapses, the core SNARE complexes contain syntaxin 3B instead of syntaxin 1A (Curtis et al., 2008, 2010). Recent researches showed that syntaxin 3B is predominantly expressed in the retina and plays a key role in ribbon synaptic vesicle exocytosis (Curtis et al., 2010). Furthermore, pathogenic variant in *STX3* (syntaxin 3) has been demonstrated to be associated with MVID and nystagmus (Julia et al., 2019), suggesting an important role of syntaxin3 in eye movement control, but detailed mechanism is still elusive. Syntaxin 3 has two major splice isoforms, syntaxin 3A (STX3A) and syntaxin 3B (STX3B) (Curtis et al., 2008). Syntaxin 3A was strongly expressed in the kidney but weakly expressed in the retina and brain, while syntaxin 3B showed strong signal in the retina, but weak signal in the cerebrum and cerebellum, and no signal in the kidney. Previous studies showed that syntaxin 3B is the major isoform of syntaxin 3 in the retina (Curtis et al., 2008, 2010), Interestingly, we found a significantly increased affinity of the binding of p.His16Arg mutant to syntaxin 3B (Figures 3F–M) and observed a significant co-localization of Munc18-1 and syntaxin 3B in mouse OPL and IPL (Supplementary Figure S6). Notably, a MUNC18-1 mutant p.R292C associated with EIEE4 and rotatory nystagmus (Stamberger et al., 2016) showed reduced interaction between MUNC18-1 and STX3B (Figures 3N,O). Based on the above, we speculate that the defect in retinal signal transduction may reflect the altered interaction between MUNC18-1 and syntaxin 3B, and this defect results in CN eventually. Further studies will be required to explore the effects of disrupted binding of MUNC18-1 to syntaxin 3B on the assembly of SNARE complexes and neurotransmitters secretion.

Given that the disrupted interaction between p.His16Arg mutant and syntaxin 3B may contribute to the impaired neurotransmission in retina, how the retinal defect in signal transduction causes congenital nystagmus? In fact, a large proportion of cases with congenital nystagmus is associated with various ophthalmology diseases, most of which are attributable to defects in visual signal transduction, such as achromatopsia (Trankner et al., 2004) and congenital amaurosis (Koenekoop, 2004), particularly the nystagmus in patients with congenital stationary night blindness was demonstrated to derive from abnormal nerve conduction in retina (Winkelman et al., 2019). In a previous study, our team also found that the FRMD7 associated CN is most likely caused by abnormal GABAergic synaptic transmission in the retina (Jiang et al., 2020). The impairment of visual transmission at infancy will impair early visual experience. It has been proposed that CN is a developmental process in which abnormal infant visual experience causes an adaptive oculomotor response that leads to nystagmus during a sensitive period of sensorimotor integration (Harris and Berry, 2006; Thomas et al., 2014). Therefore, we propose that, for the patients in this family, the impaired visual transduction since early infancy may cause abnormal visual experience that eventually results in sensorimotor integration defect and the development of nystagmus.

In summary, we identified a novel genetic locus for the AD-CN and demonstrated that the variant c.47A>G/p.His16Arg of *STXBP1*/MUNC18-1 affects neurotransmitter release in *C. elegans* and alters the interaction with syntaxin 3B. We propose that the altered interaction between MUNC18-1 and syntaxin 3B may impair neurotransmitter release at ribbon synapses, eventually leading to the abnormal retinal neurotransmission and nystagmus. Our results provide insights into the role of MUNC18-1-syntaxin 3B interaction in retinal function, and propose retina synaptic transmission as a novel target for treating these eye movement disorders.

## Data Availability Statement

The original contributions presented in the study are included in the article/Supplementary Material, further inquiries can be directed to the corresponding author/s.

## Ethics Statement

The studies involving human participants were reviewed and approved by the ethics committee of Huazhong University of Science and Technology. Written informed consent to participate in this study was provided by the participants’ legal guardian/next of kin. The animal study was reviewed and approved by Institutional Animal Care and Use Committee at Huazhong University of Science and Technology. Written informed consent was obtained from the individual(s), and minor(s)’ legal guardian/next of kin, for the publication of any potentially identifiable images or data included in this article.

## Author Contributions

JYL, XZ, CM, LZ, and SG conceived the study and designed the experiments. JYL and YL obtained financial support. JYL and YL analyzed data and wrote the manuscript. YL and CW carried out the linkage analysis and mutation screening. JYL, YL, CW, and ML supported the genetic analyses. AG and CL performed the bioinformatics analysis. LW was responsible for clinical evaluation and sample collection. LJ and YL conducted the worm tests. LJ conducted the cell culture and transfection. LJ and YL carried out immunoprecipitation, GST pull-down, western blot, immunofluorescence and microscopic analysis. CM and LZ participated in the discussion and revision of the manuscript. All authors contributed to the article and approved the submitted version.

## Conflict of Interest

The authors declare that the research was conducted in the absence of any commercial or financial relationships that could be construed as a potential conflict of interest.

## Footnotes

1 http://imagej.nih.gov/ij/

2 http://asia.ensembl.org/Multi/Tools/Blast?db=core

3 http://www.ebi.ac.uk/Tools/msa/clustalw2

4 https://embnet.vital-it.ch/software/BOX_form.html
