# Supplementary material for: p.His16Arg of STXBP1 (MUNC18-1) Associated With Syntaxin 3B Causes Autosomal Dominant Congenital Nystagmus
Source: Front Cell Dev Biol. 2020 Nov 4;8:591781. doi: 10.3389/fcell.2020.591781 (PMC7672047; doi:10.3389/fcell.2020.591781)
Supplement: Supplementary file 14 [file Data_Sheet_1.docx]

Supplementary Material

# Supplementary Figures and Tables

## Supplementary Figures


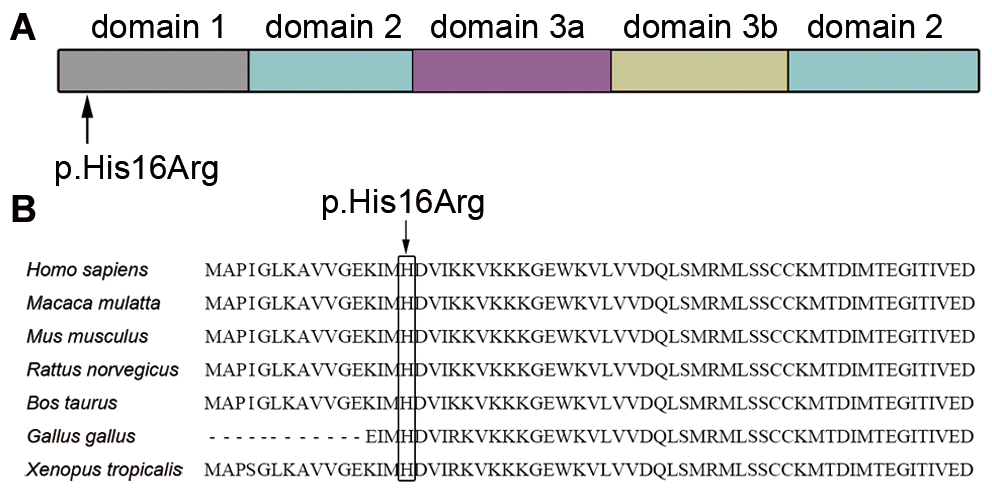


**Supplementary Figure S1.** The schematic structure of the MUNC18-1 and the alignment of amino acids around the H16 residue of the MUNC18-1 protein**. (A)** The schematic diagram of MUNC18-1 protein domain and the location of p.His16Arg in this study. **(B)** H16 amino acid residue is highly conserved from *X. tropicalis* to *H. sapiens*. The location of the variant identified in the present study is marked with arrowheads.


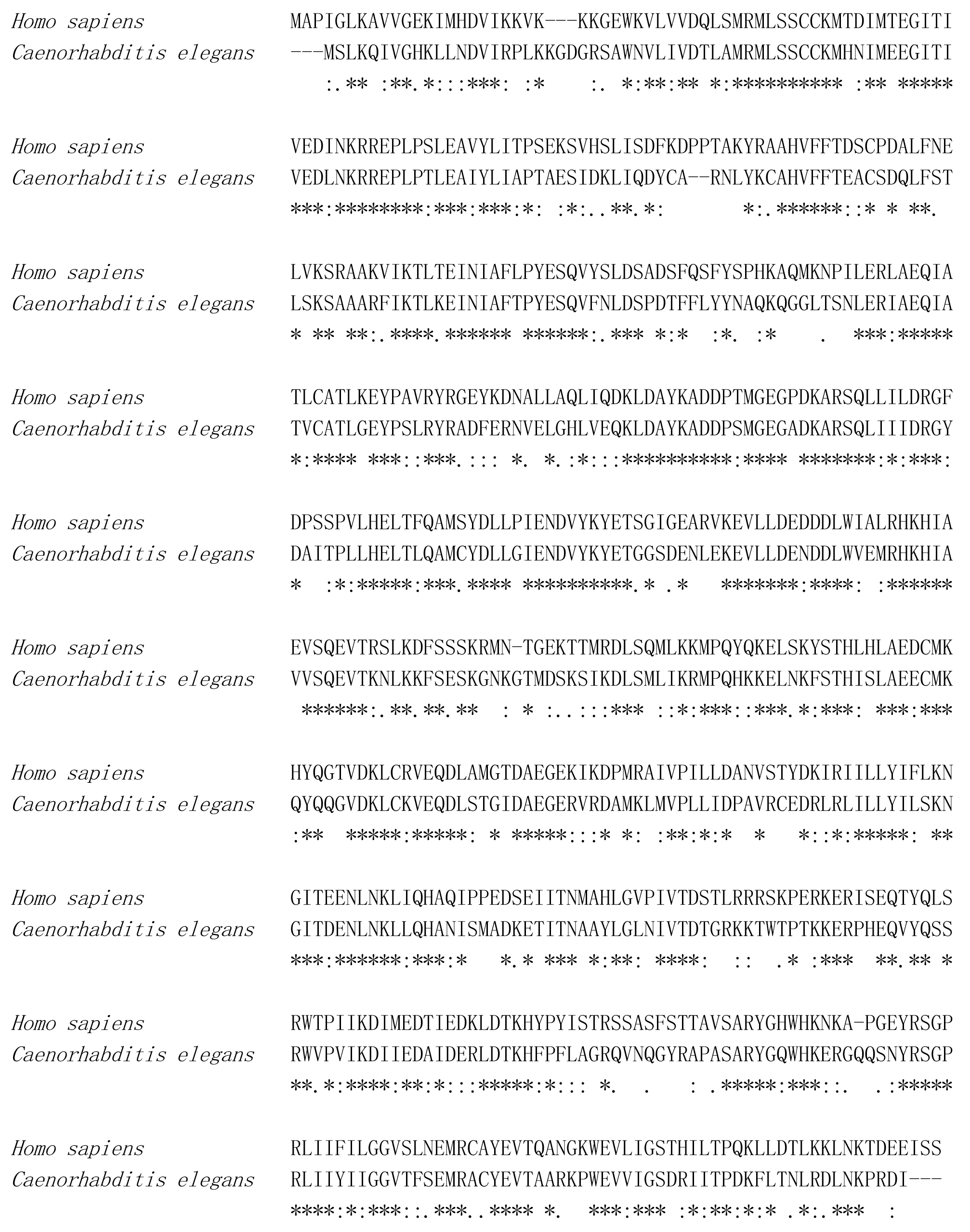


**Supplementary Figure S2.** Amino acid sequence alignment of Human (*Homo sapiens*) STXBP1/MUNC18-1 and worm (*Caenorhabditis elegans*) UNC-18 protein**.** The human STXBP1 protein shared an identity of 59% and a positivity of 75% with the worm UNC-18 protein, and the corresponding position of human STXBP1 protein His16 residue in worm UNC-18 protein is Asn13.


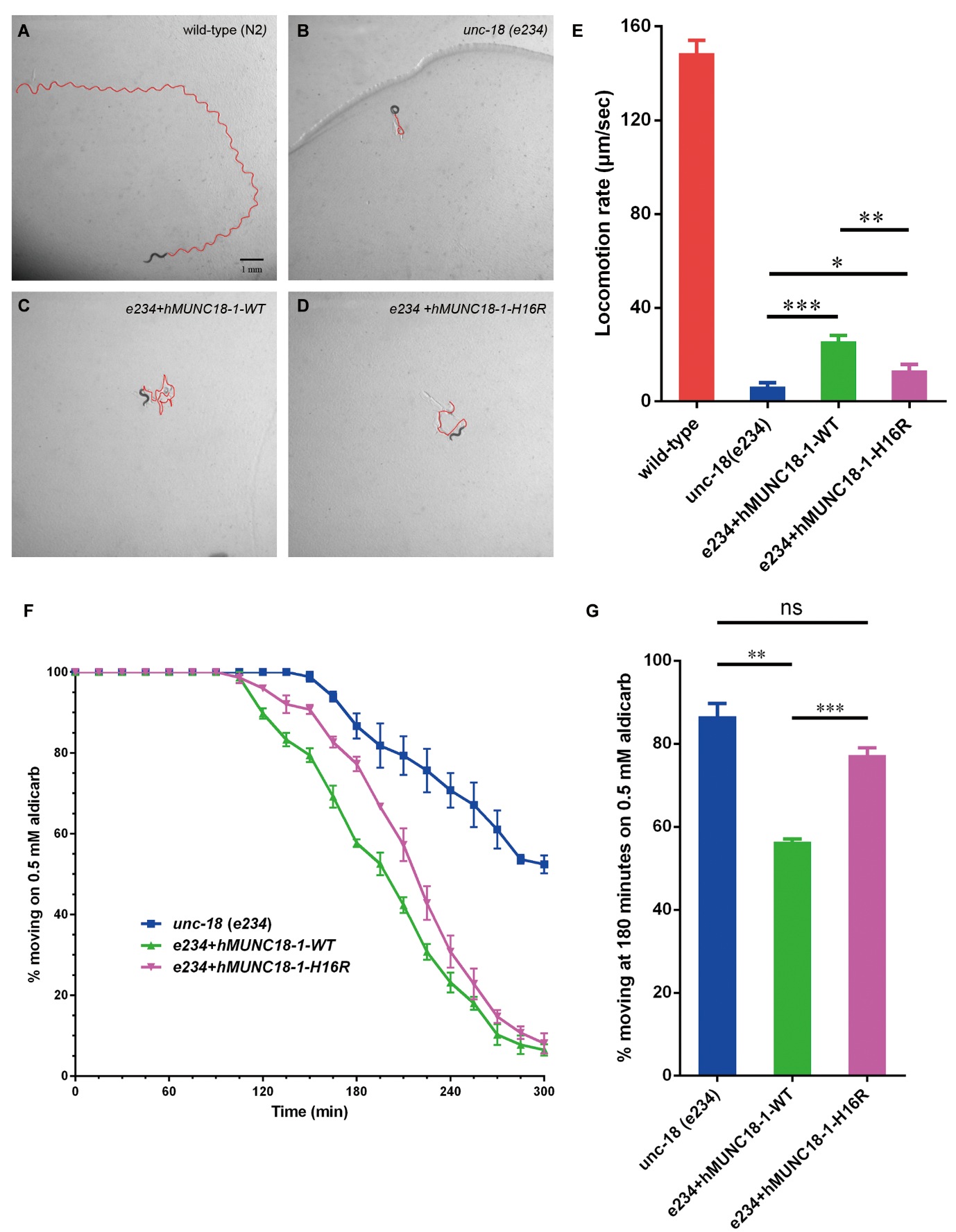


**Supplementary Figure S3.** Locomotion and aldicarb resistance in hMUNC18-1-WT and p.His16Arg mutant transgenic *unc-18(e234)* *C. elegans*. **(A-D)** The locomotion trail of wild-type **(A)**, *e234* **(B)**, *e234*+hMUNC18-1-WT **(C)** and *e234*+hMUNC18-1-H16R **(D)** worms on NGM plate in 1 minute. The traces of worms moving are outlined in red. Scale bar: 1 mm. **(E)** Both the hMUNC18-1-WT and p.His16Arg mutant significantly improve *unc-18* null worms’ motor ability, but MUNC18-1-WT transgenic *unc-18(e234)* worms show higher velocity than that of p.His16Arg mutant. Wild-type: n = 16, *e234*: n = 10, *e234*+hMUNC18-1-WT: n = 19, *e234*+hMUNC18-1-H16R: n = 14. **(F,G)** Both wild-type and p.His16Arg mutant significantly improve *unc-18* null worms’ aldicarb sensitivity (n = 3 repeats, *p* < 0.01), but the p.His16Arg mutant transgenic *e234* worms exhibit a weak rescue (n = 3 repeats, *p* = 0.055) compared to that of hMUNC18-WT transgenic *e234* worms.





**Supplementary Figure S4.** The MUNC18-1 p.His16Arg mutant shows unaltered interaction with STX1A**. (A,B)** GST pull-down assays show that p.His16Arg and p.Cys180Tyr mutants exhibit similar binding affinity to the closed form of STX1A compared to wild-type MUNC18-1, n = 3 repeats. **(C,D)** Co-immunoprecipitation assays show that p.His16Arg mutant exhibits similar binding affinity to the closed form of STX1A compared to wild-type MUNC18-1, n = 3 repeats. **(E,F)** GST pull-down assays show that p.His16Arg mutants exhibits similar binding affinity to the open form of STX1A compared to wild-type MUNC18-1, but the EIEE4-causing mutant p.Cys180Tyr exhibits reduced interaction with the open form of STX1A, which is consistent with the previous report. The experiments were repeated 3 times independently. **(G,H)** Co-immunoprecipitation assays show that p.His16Arg mutant exhibits similar binding affinity to the open form of STX1A compared to wild-type MUNC18-1, n = 3 repeats.


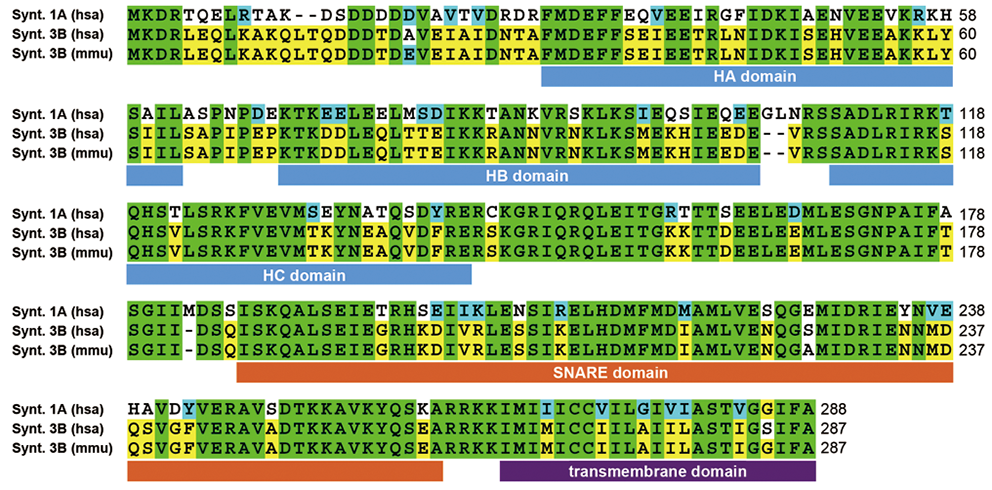


**Supplementary Figure S5.** Amino acid sequence alignment of mouse syntaxin 3B and human syntaxin 3B**.** The protein sequence of mouse and human syntaxin 3B were aligned using Clustalw2. The residues numbers are shown on the right. Identical amino acid residues are indicated with green background, while residues that are conserved only between mouse syntaxin 3B and human syntaxin 3B are labeled with yellow background. Each domain of syntaxin 3B is shown in bars under the sequences with different colors. ‘Synt.’ is the abbreviated form of syntaxin, ‘hsa’ represents *Homo sapiens*, while ‘mmu’ represents *Mus musculus*.


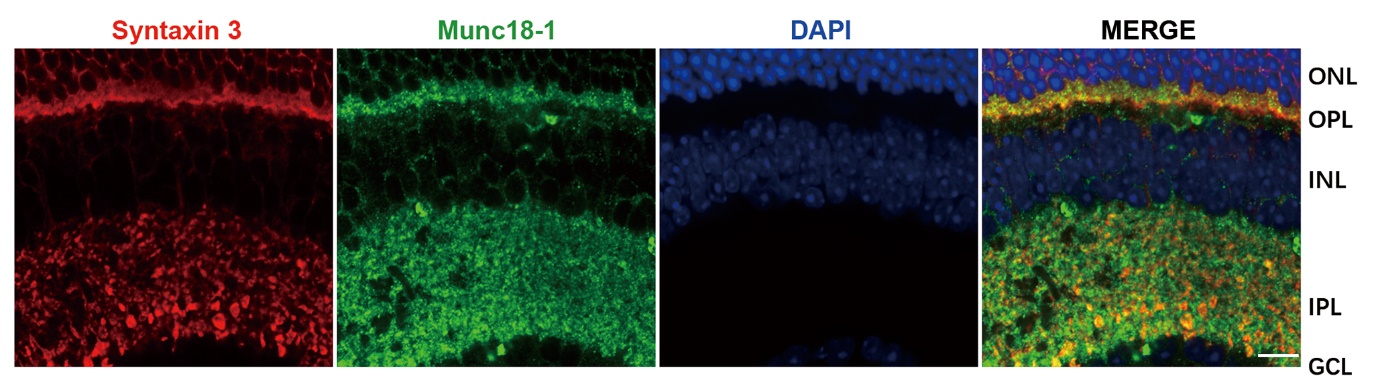


**Supplementary Figure S6.** Munc18-1 is co-localized with syntaxin 3 in mouse retina**.** Munc18-1 is partially co-localized with Syntaxin 3 in mouse retina. Retinal sections were double labeled with antibodies against Munc18-1 (green) and Syntaxin 3 (red). The nucleus (blue) was stained with DAPI. ONL, outer nuclear layer; OPL, outer plexiform layer; INL, inner nuclear layer; IPL, inner plexiform layer; GCL, ganglion cell layer. Scale bar: 20 μm.

## Supplementary Figures

**Supplementary Table** **S1.** Two-point LOD scores of microsatellite markers on 9q33.1-34.2 at different recombination fractions (θ) for the AD-CN family

| Markers | Location | LOD scores at θ | | | | | | |
| --- | --- | --- | --- | --- | --- | --- | --- | --- |
|  |  | 0.00 | 0.01 | 0.05 | 0.10 | 0.20 | 0.30 | 0.40 |
| *D9S1776* | 117.9 Mb | -∞ | 0.48 | 1.02 | 1.10 | 0.94 | 0.62 | 0.254 |
| *D9S1872* | 121.7 Mb | 4.02 | 3.95 | 3.67 | 3.30 | 2.49 | 1.60 | 0.65 |
| *D9S1116* | 122.9 Mb | 4.54 | 4.46 | 4.14 | 3.73 | 2.83 | 1.84 | 0.78 |
| *D9S1823* | 123.6 Mb | 2.45 | 2.40 | 2.21 | 1.96 | 1.43 | 0.87 | 0.33 |
| *D9S1682* | 124.9 Mb | 2.93 | 2.93 | 2.65 | 2.36 | 1.74 | 1.06 | 0.37 |
| *D9S290* | 131.4 Mb | 3.49 | 3.44 | 3.24 | 2.96 | 2.31 | 1.56 | 0.72 |
| *D9S752* | 131.8 Mb | 3.42 | 3.37 | 3.17 | 2.89 | 2.25 | 1.51 | 0.69 |
| *D9S1831* | 132.3 Mb | 2.50 | 2.45 | 2.26 | 2.02 | 1.48 | 0.88 | 0.30 |
| *D9S1861* | 133.3 Mb | 1.41 | 1.38 | 1.27 | 1.12 | 0.82 | 0.51 | 0.22 |
| *D9S164* | 136.2 Mb | -∞ | 0.62 | 1.74 | 1.98 | 1.78 | 1.27 | 0.61 |
| *D9S1826* | 138.3 Mb | -∞ | -1.85 | 0.00 | 0.58 | 0.79 | 0.59 | 0.24 |

**Supplementary Table** **S2.** Primer sequences for pathogenic variant screening of *STXBP1*

| Primer | Sequence (from 5’ to 3’) | Amplicon size (bp) |
| --- | --- | --- |
| *STXBP1*-1F | CGATTGGCGGAGGCGAGTG | 522 |
| *STXBP1*-1R | AGGGCTGGGGTGAAACATGAGG |  |
| *STXBP1*-2F | GTTATGGCTCAGTAAACCAGT | 329 |
| *STXBP1*-2R | TCCAGGAGCATTCTGTACTCT |  |
| *STXBP1*-3F | TTTGGAGTCTGATGCGGTAA | 351 |
| *STXBP1*-3R | TAGCCCTGGATTTGGTGC |  |
| *STXBP1*-4F | TTCACTCCCAAGGAACGTGGT | 389 |
| *STXBP1*-4R | ACCCCATTGTGGATTGCAT |  |
| *STXBP1*-5,6F | GGCTCCAAAGAACTGATACTGTC | 1679 |
| *STXBP1*-5,6R | AGCAAGGATGAAATGCACAC |  |
| *STXBP1*-7F | GTCCCCTGTAAACATCCCA | 669 |
| *STXBP1*-7R | ATTTGAAGTAAGAGATGCTCA |  |
| *STXBP1*-8F | TGCTCACATCTCCCCTAACCC | 310 |
| *STXBP1*-8R | GGAAAAGCAGCACTGACACA |  |
| *STXBP1*-9F | TACAGAAACTCACTTCGGCTCC | 609 |
| *STXBP1*-9R | GCACATGCCTGTAACCCCA |  |
| *STXBP1*-10F | GCTGCTGTAAGCTGAAGAGG | 262 |
| *STXBP1*-10R | AGGTGCCCAGAGTGAACG |  |
| *STXBP1*-11F | TACTGGCAGGAAAACTCAC | 435 |
| *STXBP1*-11R | CTTGAACCCAAAATGCGGAG |  |
| *STXBP1*-12,13F | TGCCCACTAGCCTTGCAAA | 1499 |
| *STXBP1*-12,13R | ATGCAAAATCCAAACGGCACA |  |
| *STXBP1*-14,15F | GCACTCAGGGCTTTTATCCAC | 1423 |
| *STXBP1*-14,15R | ACCCCTGTTATGGTTTGGTTC |  |
| *STXBP1*-16F | AGTTTGCTCCAGGTTCCC | 529 |
| *STXBP1*-16R | GCTGAGTCCTCCAAGACATAA |  |
| *STXBP1*-17F | TGCTATAACTGAGCCCTGTG | 518 |
| *STXBP1*-17R | TGAGAAGTGCGAGGAAGC |  |
| *STXBP1*-18F | TCAGCCAGCACTCACTAAGCA | 452 |
| *STXBP1*-18R | GAACCCTTTGCCCCTAGCTTC |  |
| *STXBP1*-19F | CTTTCTCATACCTCATTGGCT | 393 |
| *STXBP1*-19R | GACACATTTTCAATCGGTGTT |  |
| *STXBP1*-20F | CAACCCCAATTCTGACTCCC | 488 |
| *STXBP1*-20R | ACCAATTGCACATGAGCTTC |  |

**Supplementary Table** **S3.** Primer sequences for RFLP analysis of c.47A>G and quantitative analysis of hMUNC18-1 mRNA expression in *C. elegans*.

| Primer | Sequence (from 5’ to 3’) | Amplicon size (bp) |
| --- | --- | --- |
| c.47A>G-RFLP-F | GTTATGGCTCAGTAAACCAGT | 329 |
| c.47A>G-RFLP-R | TCCAGGAGCATTCTGTACTCT |  |
| hMUNC18-1-qPCR-F | CTACCAGCTCTCACGGTGGAC | 167 |
| hMUNC18-1-qPCR-R | GGGCCTTGTTCTTATGCCAGT |  |
| actin-worm-F | CCCAATCCAAGAGAGGTATCCTT | 187 |
| actin-worm-R | GTCATCTTTTCACGGTTAGCCTT |  |

**Supplementary Table** **S4.** Overview of the exome sequencing data production

| Exome Capture Parameters | Statistics |
| --- | --- |
| Target region (bp) | 51,543,125 |
| Raw reads | 84,663,342 |
| Raw data yield (Mb) | 7,620 |
| Reads mapped to genome | 72,920,408 |
| Reads mapped to target region | 43,192,154 |
| Data mapped to target region (Mb) | 3,309.64 |
| Mean depth of target region (X) | 64.21 |
| Coverage of target region (%) | 98.84 |
| Average read length (bp) | 89.88 |
| Rate of nucleotide mismatch (%) | 0.33 |
| Fraction of target covered >=4X (%) | 95.44 |
| Fraction of target covered >=10X (%) | 89.46 |
| Fraction of target covered >=20X (%) | 80.08 |
| Reads mapped to flanking region^1^ | 5,949,794 |
| Mean depth of flanking region(X) | 14.62 |
| Coverage of flanking region (%) | 93.81 |
| Fraction of flanking covered >=4X (%) | 71.35 |
| Fraction of flanking covered >=10X (%) | 42.74 |
| Fraction of flanking covered >=20X (%) | 22.21 |
| Gender test result | M |

^1^Flanking region refers to regions +/-200 bp on both sides of each target region.

**Supplementary Table** **S5.** PCR primer sequences for exons of low coverage in the linked region

| Primer | Sequence (from 5’ to 3’) | Amplicon size (bp) |
| --- | --- | --- |
| *ASTN2*-exon-1-F | AGCCACTGGGACTTGCACGA | 899 |
| *ASTN2*-exon-1-R | TTCCAACCACCTGTGCGACCT |  |
| *TTLL11*-exon-1-F | CTCCCGCTTCTCCTCGCCACT | 923 |
| *TTLL11*-exon-1-R | CTCGCCCGCCGACCTGACC |  |
| *ARPC5L*-exon-1-F | GCGCCCGATCCTCAGTGACA | 621 |
| *ARPC5L*-exon-1-R | ACTCAAGGGAGCACGGCCCAG |  |
| *SH2D3C*-exon-1-F | CCACCCAGGGAAGCCGAG | 608 |
| *SH2D3C*-exon-1-R | AAAAGTCCCTGATGCTAACCCC |  |
| *SH3GLB2*-exon-1-F | ATGGCGTGTACCTGCAATCATCT | 1062 |
| *SH3GLB2*-exon-1-R | CGGAGACTGAGGCTCGGAGAA |  |
| *ST6GALNAC6*-exon-1-F | CCTAGTGATCTGCCCACCTCG | 427 |
| *ST6GALNAC6*-exon-1-R | AGCCGCCTATATCTTACTACGC |  |
| *DNM1*-exon-21-F | GCATGGGCGTGGCCAGCACTG | 452 |
| *DNM1*-exon-21-R | CCGGCACCCAGAGTCCGATT |  |
| *CERCAM*-exon-1-F | CGACCGACGCAAGGAGGTGA | 973 |
| *CERCAM*-exon-1-R | TAGGTGGTGTCTGGGCAGGGAG |  |
| *NTNG2*-exon-7-F | AGGCTGTCCAAGTCGGCGTTAG | 834 |
| *NTNG2*-exon-7-R | CTGGGCGGCGGATAATACAAA |  |
| *PPP6C*-exon-2-F | TCCCGTTTCTGAGTATGTTG | 384 |
| *PPP6C*-exon-2-R | TAATTCCACCTAGTCGGGAG |  |
| *RALGDS*-exon-6-F | CCCAGATGAGCCTCAGACCAC | 838 |
| *RALGDS*-exon-6-R | CCTGCTGAGCTTCCTGTCTGT |  |
| *RALGDS*-exon-1-F | CACATTTCAGCCCTACGCAACA | 945 |
| *RALGDS*-exon-1-R | ACAGGCCACCGATGTAAACAGG |  |
| *PIP5KL1*-exon-5-F | CCTGGCGGAGGAGGACTATCA | 878 |
| *PIP5KL1*-exon-5-R | TCGGCTTCACCCACCTTCTTT |  |
| *PIP5KL1*-exon-2-F | CTCACAAGTAGTGGAAGTGGGACT | 357 |
| *PIP5KL1*-exon-2-R | AGCCAGACAGGGCATCAGC |  |
| *LHX2*-3UTR-F | TTTCTAATGACTCGCAACCC | 752 |
| *LHX2*-3UTR-R | GCATCTCTGTAATCCGTCCA |  |
| *CDK5RAP2*-exon-16-F | TTTCAGCCATGTAAATTACGC | 503 |
| *CDK5RAP2*-exon-16-R | GAAACCCCGTGTATGCTT |  |
| *GSN*-exon-1-F | TTTGCTGGAGGTGTTAGGTGC | 772 |
| *GSN*-exon-1-R | ATTCAGGAGACAGGAGGTAAGATG |  |
| *DAB2IP*-exon-3-F | GCGTCGCCAAGGCAACAGC | 757 |
| *DAB2IP*-exon-3-R | GCAGAGGCCCCACTTCCGTC |  |
| *CDK9*-exon-1-F | TAGTCGGCTCTTCCCGTCTCGC | 946 |
| *CDK9*-exon-1-R | CCTTCCCTCAGTCCCTTCTCCTCA |  |
| *CIZ1*-exon-13-F | CCCAAACTCACAGCGGGTC | 486 |
| *CIZ1*-exon-13-R | CCAAATTTCAGTCAGGCTTTGCAT |  |
| *CIZ1*-exon-7-F | AGTGCTGGGTTTACAGATGCG | 1067 |
| *CIZ1*-exon-7-R | CACAGGCTCTGGCAAGGTC |  |
| *CIZ1*-exon-1-1F | GGGCTGTTGGCAGTGTCTG | 606 |
| *CIZ1*-exon-1-R | AAGGCTTCTCACCTCCATTCT |  |
| *PKN3*-exon-1-F | TTAGCCCACATTACACCAACAACG | 822 |
| *PKN3*-exon-1-R | GTGCAAGGTCCAGGAACCACC |  |
| *LAMC3*-exon-1-F | CTATGCGGAGCCATCTCACAA | 1011 |
| *LAMC3*-exon-1-R | GCACCCAGCCTAATTCCTATTTC |  |
| *LAMC3*-exon-9-F | GGGGTCTTCAGGCTGTCTT | 401 |
| *LAMC3*-exon-9-R | ATGCGGCAAGTTGTTTAGC |  |
| *POMT1*-exon-15-F | ACCAGTTTATCCCCGTGCAAG | 575 |
| *POMT1*-exon-15-R | CCTTAAGACACCCTGGCGATG |  |
| *DENND1A*-exon-22-F | CCACTGGCATAAAGGGACGAG | 1667 |
| *DENND1A*-exon-22-R | TGGGATGGGCACTCAGGAA |  |
| *DENND1*-exon-1-F | GGCGGCTGTGAGTGGAGGTT | 809 |
| *DENND1*-exon-1-R | CCCCACAGTTGAAGGACCATTTT |  |
| *GAPVD1*-exon-6-F | TTTTACCACTCTTGTGCAT | 371 |
| *GAPVD1*-exon-6-R | TATAAGCCACCCAACCTC |  |
| *GAPVD1*-exon-13-F | TTTTCTGGTGAGATTGGTGGTG | 470 |
| *GAPVD1*-exon-13-R | TGGGATTATGGGCGTGAG |  |
| *MEGF9*-exon-1-F | GGCTTCCCATTGGTTAGGCG | 1189 |
| *MEGF9*-exon-1-R | ACTATGCGGGCTTTCAAACTTCC |  |
| *CRB2*-exon-1-F | GAGGTGGAGCAGCCCAGGAG | 514 |
| *CRB2*-exon-1-R | CCCCAACCCCGAAACTTACA |  |
| *CRB2*-exon-4-F | GTGCCACGACCTGGTCAACG | 589 |
| *CRB2*-exon-4-R | GCGCCCGGCCTAGTCTTACT |  |
| *CRB2*-exon-6-F | AAGCGGTCAGCCCATGTCCA | 569 |
| *CRB2*-exon-6-R | AAGACCATCCCTCCACACGTT |  |
| *CRB2*-exon-10-F | ACATTGGGAGTAGAAGTGAGGGTG | 1178 |
| *CRB2*-exon-10-R | GCTGTTTATCTGAAATTCCAGGCT |  |
| *GOLGA2*-exon-21-F | GAACTCCCATGCTGATCGGTA | 653 |
| *GOLGA2*-exon-21-R | TCCTTCAGCTGCCCACGTA |  |
| *GTF3C4*-exon-1-F | GCCTGAGGGGAGAAAACCG | 814 |
| *GTF3C4*-exon-1-R | GGGGTGGAGTCTCAGAAAGCA |  |
| *GTF3C5*-exon-11-F | GTGCCCGTCAAACTACAGCTT | 1042 |
| *GTF3C5*-exon-11-R | CCTCACCCAACCCGACCAC |  |
| *NR6A1*-exon-1-F | ACTCCAACTTCCAGCGTGCCC | 557 |
| *NR6A1*-exon-1-R | TCCACCCTGAGCGAGACCG |  |
| *NUP188*-exon-1-F | AACACTACTGCCGCCTCTGCC | 1026 |
| *NUP188*-exon-1-R | CTCCGCTCAACGCGAAACA |  |
| *SCAI*-exon-1-F | ATTGGGAGATTTCAGGGCTTGTT | 940 |
| *SCAI*-exon-1-R | CACCTACTCATTGTCTGCCTCGTC |  |
| *FAM129B*-exon-1-F | GGGGCACTGTTCAGGGTAGAGGA | 1185 |
| *FAM129B*-exon-1-R | CCCAAAGCTGGTAGCGGATGG |  |
| *LOC389791*-F | CCGCTTCCTACTGTGACCCGAAC | 1094 |
| *LOC389791*-R | CAGCAGGCCAAACACGGACCC |  |

**Supplementary Table** **S6.** The variations located in the linked region (9q33.1-q34.2) with MAF (minor allele frequency) no more than 0.01 in the gnomAD database.

| **Position** | **Ref** | **Alt** | **GeneName** | **cDNA**  **change** | **protein**  **change** | **rsid** | **allele frequency in gnomAD** |
| --- | --- | --- | --- | --- | --- | --- | --- |
| chr9:120476549 | G | A | *TLR4* | c.2143G>A | p.G715S | rs199930089 | 0.0001086 |
| chr9:125437611 | T | C | *OR1L3* | c.203T>C | p.F68S | rs143061509 | 0.002241 |
| chr9:125486717 | G | A | *OR1L4* | c.449G>A | p.C150Y | rs76170289 | 0.002623 |
| chr9:125563110 | C | T | *OR1K1* | c.709C>T | p.R237W | rs147003720 | 0.0007456 |
| chr9:130413891 | A | G | *STXBP1* | c.47A>G | p.H16R | rs571127140 | 0.000003978 |
| chr9:131020815 | C | A | *GOLGA2* | c.2127G>T | p.E709D | rs62587120 | 0.00002008 |
| chr9:131600356 | G | A | *CCBL1*^1^ | c.412C>T | p.R138C^1^ | rs528324992 | 0.0001388 |
| chr9:133231243 | T | C | *HMCN2* | c.5252-2T>C | SpliceSite | not found | not found |
| chr9:133967099 | C | T | *LAMC3* | c.698C>T | p.P233L | rs182704192 | 0.002248 |
| chr9:134459805 | G | A | *RAPGEF1* | c.2809-6C>T | SpliceSite | rs184288936 | 0.0008671 |
| chr9:135388689 | A | C | *C9ORF171*^2^ | c.568A>C | p.N190H^2^ | rs961042204 | 0.00002697 |

^1^The variation c.412C>T/p.R138C was not conserved through phylogenetic analysis, even the rat (*Rattus norvegicus*) harbors the cysteine at the same residue.

^2^The variation c.568A>C/p.N190H of C9ORF171 was not segregated with the nystagmus phenotype in the family, which indicates the variation may lie outside the boundaries of the linked interval.
